# A Systematic Review of Fitness Apps and Their Potential Clinical and Sports Utility for Objective and Remote Assessment of Cardiorespiratory Fitness

**DOI:** 10.1007/s40279-019-01084-y

**Published:** 2019-03-01

**Authors:** Adrià Muntaner-Mas, Antonio Martinez-Nicolas, Carl J. Lavie, Steven N. Blair, Robert Ross, Ross Arena, Francisco B. Ortega

**Affiliations:** 1 GICAFE “Physical Activity and Exercise Sciences Research Group”, University of Balearic Islands, Balearic Islands, Spain; 2 PROFITH “PROmoting FITness and Health through physical activity” Research Group, Department of Physical Education and Sports, Faculty of Sport Sciences, University of Granada, Granada, Spain; 3 Chronobiology Research Group, Department of Physiology, Faculty of Biology, University of Murcia, Campus Mare Nostrum, IUIE, IMIB-Arrixaca, Murcia, Spain; 4 Ciber Fragilidad y Envejecimiento Saludable (CIBERFES), Madrid, Spain; 5 Department of Cardiovascular Diseases, John Ochsner Heart and Vascular Institute, Ochsner Clinical School, The University of Queensland School of Medicine in New Orleans, New Orleans, LA USA; 6 Department of Exercise Science, Arnold School of Public Health, University of South Carolina, Columbia, SC USA; 7 School of Kinesiology and Health Studies, Queen’s University, Kingston, ON Canada; 8 Department of Physical Therapy, College of Applied Health Sciences, University of Illinois at Chicago, Chicago, IL USA; 9 Department of Biosciences and Nutrition at NOVUM, Karolinska Institutet, Huddinge, Sweden

## Abstract

**Background:**

Cardiorespiratory fitness (CRF) assessment provides key information regarding general health status that has high clinical utility. In addition, in the sports setting, CRF testing is needed to establish a baseline level, prescribe an individualized training program and monitor improvement in athletic performance. As such, the assessment of CRF has both clinical and sports utility. Technological advancements have led to increased digitization within healthcare and athletics. Nevertheless, further investigation is needed to enhance the validity and reliability of existing fitness apps for CRF assessment in both contexts.

**Objectives:**

The present review aimed to (1) systematically review the scientific literature, examining the validity and reliability of apps designed for CRF assessment; and (2) systematically review and qualitatively score available fitness apps in the two main app markets. Lastly, this systematic review outlines evidence-based practical recommendations for developing future apps that measure CRF.

**Data Sources:**

The following sources were searched for relevant studies: PubMed, Web of Science^®^, ScopusTM, and SPORTDiscus, and data was also found within app markets (Google Play and the App Store).

**Study Eligibility Criteria:**

Eligible scientific studies examined the validity and/or reliability of apps for assessing CRF through a field-based fitness test. Criteria for the app markets involved apps that estimated CRF.

**Study Appraisal and Synthesis Methods:**

The scientific literature search included four major electronic databases and the timeframe was set between 01 January 2000 and 31 October 2018. A total of 2796 articles were identified using a set of fitness-related terms, of which five articles were finally selected and included in this review. The app market search was undertaken by introducing keywords into the search engine of each app market without specified search categories. A total of 691 apps were identified using a set of fitness-related terms, of which 88 apps were finally included in the quantitative and qualitative synthesis.

**Results:**

Five studies focused on the scientific validity of fitness tests with apps, while only two of these focused on reliability. Four studies used a sub-maximal fitness test via apps. Out of the scientific apps reviewed, the *SA-6MWTapp* showed the best validity against a criterion measure (*r* = 0.88), whilst the *InterWalk* app showed the highest test–retest reliability (ICC range 0.85–0.86).

**Limitations:**

Levels of evidence based on scientific validity/reliability of apps and on commercial apps could not be robustly determined due to the limited number of studies identified in the literature and the low-to-moderate quality of commercial apps.

**Conclusions:**

The results from this scientific review showed that few apps have been empirically tested, and among those that have, not all were valid or reliable. In addition, commercial apps were of low-to-moderate quality, suggesting that their potential for assessing CRF has yet to be realized. Lastly, this manuscript has identified evidence-based practical recommendations that apps might potentially offer to objectively and remotely assess CRF as a complementary tool to traditional methods in the clinical and sports settings.

**Electronic supplementary material:**

The online version of this article (10.1007/s40279-019-01084-y) contains supplementary material, which is available to authorized users.

## Key Points

**Table:** 

The validity and reliability of existing and/or under-development fitness apps should be further investigated.
Physiological signals should be incorporated into fitness apps, such as heart rate measures using a smartphone camera, during or after exercise testing.
There is a need to develop interoperable fitness apps (e.g., different languages, apps integrated into both app markets, data that is device-independent).
Fitness apps should incorporate evidence-based cut-points of CRF, allowing interpretation of fitness testing results.

## Introduction

Cardiorespiratory fitness (CRF) is a powerful marker of cardiovascular (CV) health [1–6]. Despite the strong existing evidence linking CRF to CV health, the recent eHealth tools developed to assess CV disease (CVD) risk on the basis of multiple risk factors does not include CRF as a measure [7]. Further, maximal oxygen uptake (*V*O_2max_) is an objective measure of CRF and has been considered a key indicator of sports performance [8–10]. In fact, *V*O_2max_ assessment has been historically recommended in both the clinical and sports settings by the American College of Sports Medicine (ACSM) and the American Heart Association (AHA) [11, 12]. Since an incremental maximal or submaximal exercise test is not always possible in clinical or field settings, in part due to feasibility concerns with its routine measurement (e.g., time needed, expensive equipment, expertise required, etc.), estimations of CRF using non-exercise algorithms have a pragmatic importance that may enhance CVD risk and sports performance prediction [13–16]. However, the rapid development in smartphone technology might provide a novel alternative to non-exercise algorithms to estimate CRF (i.e., *V*O_2max_) in the present and future. Such an approach could be useful and meaningful from a clinical point of view as well as from a sports and training landscape.

### Usefulness of Smartphone Apps in Clinical and Sports Context

Technological advancements have led to increased digitization within healthcare and sports [17, 18]. The emergence of available smartphone applications (apps) in Google Play and the App Store (iTunes) in September 2008 and June 2009, respectively, have contributed to a better understanding of human health by allowing us to gather vast amounts of medical and fitness data [19]. Specifically, some improvements in app technology (e.g., a built-in camera for heart rate assessment, accelerometers, etc.) have opened new opportunities for collecting relevant information in the clinical and sports settings.

In fact, successful examples of clinicians and scientists using apps that allow for a flood of new information for better management of a patients’ CV health are already available [20]. More specifically, the usefulness of apps in clinical practice is supported by current reviews of CV mHealth (healthcare practice supported by mobile devices), which have outlined the potential of these apps to improve access to a large number of people living far from clinical centers, reduce costs, and enhance health outcomes for CVD management [21–23]. Within this context, the use of apps for telemedicine purposes has demonstrated their potential and effectiveness for remote monitoring of clinical parameters, such as CVD risk factors [24]. An example of this practice is the AliveCor Kardia device, a clinically validated smartphone-based electrocardiogram recording [25]. A recent randomized controlled trial has examined the assessment of remote heart rhythm in 1001 ambulatory patients ≥ 65 years of age at increased risk of stroke who were using this device. The results highlight that this approach was significantly more likely to identify incident atrial fibrillation than routine care over a 12-month period [26]. If these innovative clinical practices are viable with other vital signs, it can be speculated that apps may hold utility in detecting patients with low CRF levels, and in turn allow for a more accurate determination of CVD risk [27].

The use of apps to collect data has also drawn widespread attention among sports professionals and exercise scientists. In fact, some apps have already been developed to collect physiological, kinanthropometric, and sports performance data [28]. The use of apps for data collection is likely the most popular in recreational sporting activities, although they are also utilized in a higher performance sporting context [29]. In high-performance sports, the expertise required to quantify an athlete’s physical performance with traditional methods is often expensive and non-user-friendly, especially for trainers [28]. However, apps hold great potential by making physical performance measurements for coaches and trainers more affordable in field conditions. A popular recreational example is the various apps designed for tracking distance or pace during endurance sports [30–32]. A real-world app is *Strava*, commonly used for individuals practicing recreational endurance activities [32]. Among the most attractive *Strava* features is its ability to track all aspects of logged physical activities (e.g., distance, pace, watts, heart rate) and its capability to analyze them on a per-minute basis. Likewise, in a competitive sporting context, there are already validated apps aimed at coaches for assessing sports performance data such as sprint mechanical outputs [33] and running technique [34].

### Usefulness of Fitness Apps for Assessing CRF in Clinical and Sports Context

Fitness apps might provide a valuable opportunity for assessing CRF, bringing the laboratory into the pocket, and making fitness assessments feasible as part of routine clinical care [35]. Furthermore, using apps for CRF self-assessment as part of the clinical workflow might provide clinicians with health information difficult to collect during brief patient visits, furthermore allowing integration of this data into the electronic health record and aid in ongoing care [35, 36]. Despite the plethora of existing traditional approaches to CRF assessment, some barriers hamper its use in clinical practice. For instance, the correct selection of a CRF protocol according to a person’s individualized exercise or functional capacity can be challenging at times [37]. In addition, making this selection often requires professionals with advanced training in CRF measurement not always available in the clinical setting. Another hurdle to performing clinical CRF assessments is the use of specialized equipment (e.g., ECG, pulse oximetry, accelerometers, etc.) that may not be available. In this context, an app-based approach could overcome these challenges associated with traditional approaches, allowing for broader application of CRF assessments. For instance, a valid and reliable fitness app might assist health professionals in the selection of the optimal CRF assessment protocol and integrate the measurement of physiological signals to more objectively assess CRF. Also, these apps could be useful for screening programs identifying individuals with higher versus lower CVD risk based on the app-assessed CRF level. As a current clinical example, the *MyHeart Counts* app has demonstrated real-world feasibility in assessing CRF on a large scale, incorporating this assessment into the broader evaluation of CV health [36, 38].

The assessment of CRF for sports and training purposes is also an important function of health fitness professionals [11]. In fact, the use of apps for assessing other physical fitness components such as muscular fitness is a current practice in the sports field. As examples, the *My Jump* and *PowerLift* apps have shown scientific validity and reliability for measuring distinct aspects of muscular performance [39, 40]. Further, both apps are being used by many sports professionals in field settings. However, health fitness professionals are demanding valid tools for the remote and objective assessment of CRF. In this context, the usefulness of apps for CRF assessment might provide coaches with additional data difficult to obtain in field settings with traditional approaches. Specifically, an app-based approach for determining CRF in sports could add value to exercise prescription and monitoring training. For instance, with exercise prescription, these apps could be used before a training session to adjust intensities for training in the appropriate intensity zone and obtain the best physiological adaptations for athletes, reducing the risk of overtraining. In this context, an example is the *HRV4Training* app that provides analyses on the relationship between physiological parameters (taking measures of heart rate and heart rate variability), training and performance. In brief, the *HRV4Training* app estimates acute heart rate variability changes in response to acute stressors that affect an athlete’s acute physiology. This data can be used for determining athlete fatigue and thus modifying the training program of the athlete from day to day and in real time. Likewise, apps assessing CRF might hold value in monitoring cardiovascular performance changes throughout a conditioning program and tracking injury risk factors affecting cardiovascular functioning. Also, CRF measurements with fitness apps could advance the research field, making CRF assessment more affordable and potentially self-administered by the athletes.

### Purpose

The purpose of this review article is to facilitate a scientific discussion about the new opportunities that advances in apps offer, such as the ability to objectively and remotely assess CRF as a complementary tool to traditional methods for estimating CVD risk, as well as to assess CRF for improving performance in sports and training. To address the purpose of this review and provide evidence-based practical recommendations for researchers, clinicians, and sports professionals, the following original two-pronged approach has been employed: (1) a systematic review of the available scientific literature, examining the validity and reliability of apps designed for CRF assessment, and (2) a systematic analysis of apps estimating CRF and stored within the two major app markets (Google Play and App Store).

## Methods

The search strategy, criteria, and related terms used in both the scientific literature and the app market search are presented in Supplemental Tables 1–5 in the electronic supplementary material (ESM).

### Scientific Literature Search

#### Literature Search Strategy and Study Selection Process

**Fig. 1:**
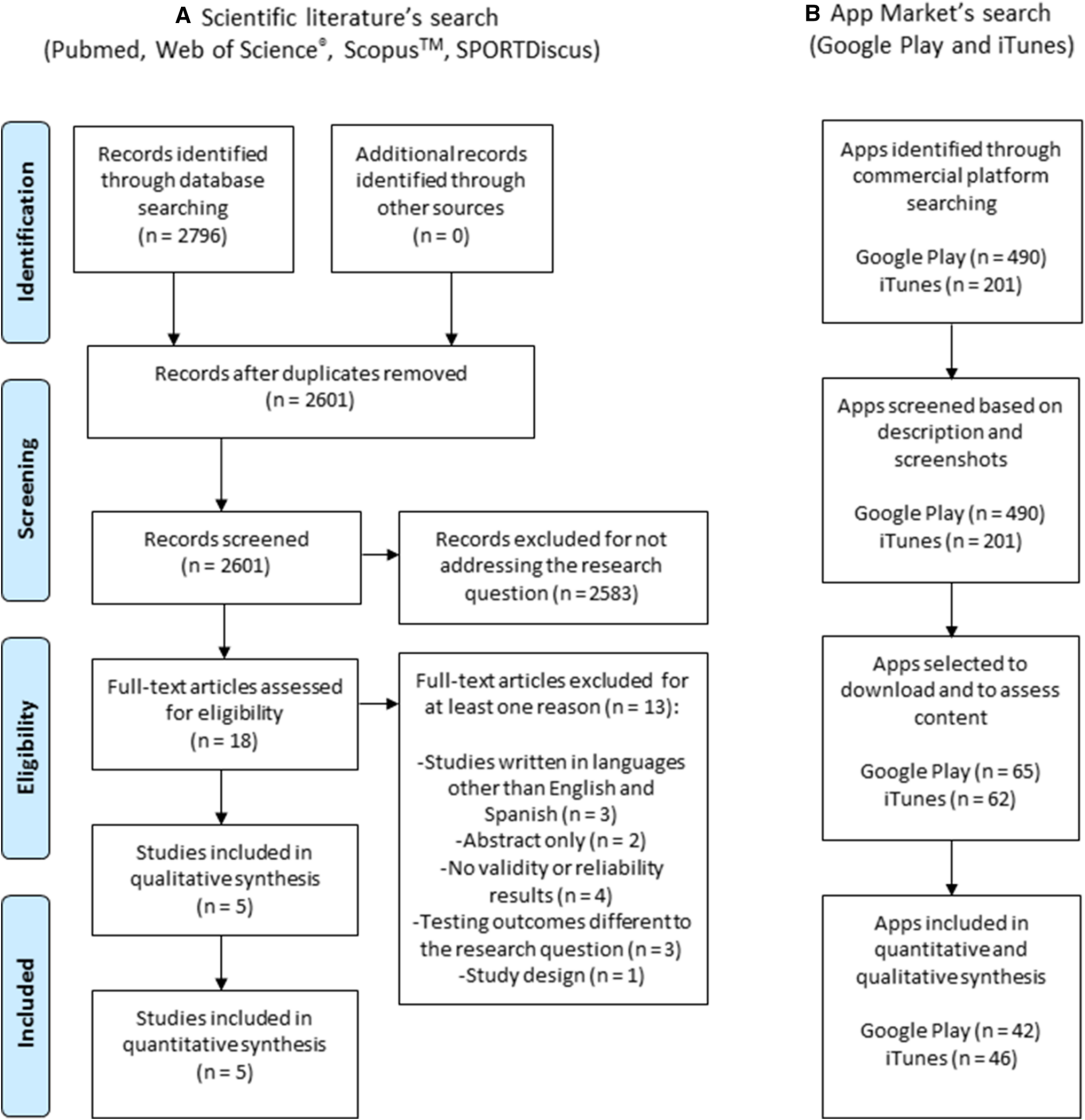
Flow chart: an overview of the review process for the scientific literature search and the app market search

The literature search was carried out according to the PRISMA (Preferred Reporting Items for Systematic Reviews and Meta-Analyses) statement [41]. The search included four major electronic databases (i.e., PubMed, Web of Science^®^, Scopus™, and SPORTDiscus) and the timeframe was set between 01 January 2000 and 31 October 2018. Even though the two main markets, Google Play, and the App Store, were launched between 2007 and 2008, native apps (apps developed for use on a specific device) began to appear commercially around 2000; therefore, the timeframe was set based on the emergence of the first native apps. For searching in PubMed, we used Medical Subject Heading (MeSH) terms and a combination of relevant keywords in the field (see Supplemental Table 1 in the ESM). The same search strategy and the combination of terms were repeated in Web of Science^®^, Scopus™, and SPORTDiscus, but without using MeSH terms (see Supplemental Tables 2–4 in the ESM). The reference lists of included articles were also searched for additional studies. Included were studies that examined the validity and/or reliability of apps for assessing CRF using a field-based fitness test. Studies were excluded according to the following criteria: (1) studies written in languages other than English and Spanish, and (2) studies from which we could not access the full text. The selection procedure of the 2796 articles initially identified was undertaken following a two-step approach: (1) screening based on the title and abstract; and (2) search of the full text of the articles selected in the previous step. The first two authors (AM-M and AM-N) independently performed the study selection process and disagreements were resolved in a consensus meeting. The selection process for scientific studies is illustrated in Fig. 1a.

#### Data Collection Process

For selected articles, data extraction was undertaken by the first author (AM-M) and the second author (AM-N) confirmed accuracy. A standardized data extraction form was utilized and is presented in Table 1. Data extracted included details on source (authors, year, name of the fitness app, and market platform), information about the participants (age, sample size, and gender), fitness test examined, criterion measure used as gold standard, main outcomes studied, statistical methods, and the validity and/or reliability of results.

**Table 1:** Scientific studies examining the validity/reliability of apps for assessing cardiorespiratory fitness

Study Name of the app (market platform)	Age (years) ± SD Sample size (male, female)	Fitness test	Measure criterion	Main outcomes	Statistical method	Validity and/or reliability results
Altini et al. [44] HRV4Training (Google Play and iTunes)	*Laboratory data* 25.0 ± 6.2 48 (22, 26)	Prediction of *V*O_2max_ taking anthropometrics data, physiological data, and training data	Incremental test on a cycle ergometer	*V*O_2max_	Correlation coefficient (*r*) Bland–Altman plots RMSE	Validity (*r* = 0.72–0.8 between estimated and measured *V*O_2max_)
Brinkløv et al. [46] InterWalk app (iTunes)	64.2 ± 5.9 27 (9, 18)	InterWalk Fitness Test (sub-maximal)	Indirect calorimetry with a graded walking test protocol on a treadmill	*V*O_2peak_ and *V*O_2max_	The correlation coefficient (*r*), Leave-one-out cross-validation, ICC, Bland-Altman plots, minimal detectable change	*V*O_2peak_ prediction of the algorithm (*R*^2^) was 0.60 and 0.45 and the test–retest ICC was 0.85 and 0.86 when the smartphone was placed in the pockets of the pants or jacket, respectively
Brooks et al. [45] SA-6MWTapp (not available in markets)	*Home validation* (*Phase III*) 54 ± 19 19 (6, 13)	6-Minute walk test (sub-maximal)	In-clinic known steps count and measured distance	Step count and distance walked	Paired *t* test, 3-knot restricted cubic splines, tenfold cross-validation, ICC, CV	*r* = 0.88 between in-clinic distance and SA-6MWTapp. The CV during home validation trials was 4.6%
Capela et al. [48] 2–6MWT app (not available in markets)	5 participants	2-minute walk test (sub-maximal)	Digital video and measuring tape	Distance walked, total number of steps, number of steps per walkway length, cadence, step time, stride time, step time symmetry	The systematic error between studied outcomes and the gold standard	Foot strike time was within 0.07 seconds when compared with gold standard video recordings. The total distance calculated by the app was within 1 m of the measured distance
Capela et al. [47] TOHRC Walk Test (Google Play)	10 males (40.6 ± 15.9 years) 5 females (38.8 ± 9.7 years)	6-Minute walk test (sub-maximal)	Digital video and measuring tape	Distance walked, total number of steps, number of steps per walkway length, cadence, step time, stride time, step time symmetry	The systematic error between studied outcomes and the gold standard	The average error in the calculated distance was 0.12%. The average difference between smartphone and gold standard foot strike timing was 0.014 ± 0.015 s

*CV* coefficient of variation, *HR* heart rate, *HRV* heart rate variability, *ICC* intraclass correlation coefficient, *RMSE* root mean square error, *VO*_*2*_ oxygen consumption

#### Assessment of Methodological Quality

The risk of bias within scientific studies finally included was assessed by using some elements of the Cochrane Collaboration’s tool for assessing the risk of bias [42]. Specifically, the domains analyzed in the present systematic review were detection bias, attrition bias, reporting bias, and other bias.

### App Markets Search

#### Apps Search Strategy and Selection Process

The app market searches were conducted in the Spanish Google Play and App Store. However, we also performed manual searches for other relevant apps in the United States app market. Apps from Google Play and App Store were screened in October 2018. The apps were identified by introducing keywords into the search engine of each app market without specified search categories (see Supplemental Table 5 in the ESM). The inclusion criteria were fitness apps that assess CRF with physiological signals, integrated algorithms, and/or fitness apps serving as a simple CRF calculator. However, those apps not in English or Spanish, not readily accessible or unable to be downloaded were excluded. The selection procedure of the apps on both app markets was carried out by the first author (AM-M) using the following two-step method: (1) screening apps based on description and screenshots; and (2) 1 week after the first search, a second screening was performed following the identical selection process. Apps identified in the two steps were selected for download and content assessment. The selection process for apps is illustrated in Fig. 1b.

#### Data Collection Process and Qualitative Assessment

Selected apps were downloaded to a smartphone (Apple or Android software) by AM-M and AM-N. In those cases in which an app had a free and a paid (premium/more advanced) version, both apps were downloaded and assessed. Further, the data extraction was undertaken using the following two-step method: (1) AM-M and AM-N independently assessed five apps using two qualitative instruments (explained below); this first step was to ensure that both reviewers used the same criteria to fill both qualitative instruments; and (2) AM-M extracted and scored information of all apps stored on App Store and AM-N did the same with apps stored in Google Play.

Two qualitative assessments were carried out with apps ultimately included in the systematic review. First, the Mobile App Rating Scale (MARS) was used to rate app quality [43]. The selected apps were tested for at least 10 min and rated with this scale. In brief, the MARS has 29 items measured on a 5-point scale grouped into six domains (engagement, functionality, aesthetics, information, subjective and perceived impact). An overall score was computed as a MARS mean considering four domains (engagement, functionality, aesthetics, information). Second, a standardized instrument was developed specifically for this review to evaluate some features of the apps included (score, ratings, downloads, price, fitness test, test instructions, heart rate measurement, GPS, maximal or peak oxygen consumption (*V*O_2_) estimation, external equipment needed, historic measurement, export results, prompt self-monitoring of behavioural outcome, social network, reference values, scientific validation, multiple user, language, and last update date). Twelve of these items were rated as ‘yes’ or ‘no’, for instance, if an app included CRF reference values, the app was rated as ‘yes’ for this item. The sum of total ‘yes’ responses per app was computed as an average of the quality of the apps. Pearson correlations were used to examine the relationships between the cost of apps, their features, and MARS mean score. All statistical analyses were conducted using IBM SPSS Statistics version 22.4 (Armonk, NY, USA), with significance levels set at *p *< 0.05.

## Results

### Scientific Literature Results

#### Studies’ Characteristics

Figure 1 illustrates the flow chart of the scientific review (according to PRISMA), as well as the flow chart of the review of the current app markets. The scientific studies selected are summarized in Table 1. The results of the scientific literature’s search revealed that there were five studies [44–48] published in peer-reviewed journals, of which only three apps were available to be downloaded on commercial platforms (*HRV4Training*, *InterWalk* app, and *TOHRC Walk Test*). *HRV4Training* [44] was the only app stored in both app markets (Google Play and App Store). All the included apps were available in English, except for the *InterWalk* app, which was only available in Danish [46]. Four studies [45–48] included < 20 participants, and one study [44] included 48 individuals. Three studies included healthy adults [44, 47, 48], whereas Brinkløv et al. [46] included participants with type 2 diabetes mellitus and Brooks et al. [45] included those with congestive heart failure and pulmonary hypertension. The 6-minute walk test was used in two studies [45, 47]. Two studies [45, 46] were adjudicated to be of low risk of bias and three [44, 47, 48] were considered to have a high risk of bias. The criteria for high risk of bias were (1) the study failed to include the complete methodology to assess validate/reliability of the fitness test with the app; and (2) the study did not entirely report the results or analysis methods of the outcomes studied.

#### Fitness Test Assessment Methods

Altini et al. [44] used information from three sets of predictors (models hereafter) for quantifying CRF: (1) anthropometric data (body mass index, age, and gender) taken from the *HRV4Training* app; (2) physiological data (morning heart rate and heart rate variability) acquired with the *HRV4Training* app at rest conditions plus model 1; and (3) training data measured as the ratio between running speed (retrieved from the *Strava* app and linked to *HRV4Training*) and morning heart rate (retrieved from *HRV4Training*) plus model 1. The criterion CRF was determined as *V*O_2max_, by means of cardiopulmonary exercise testing (CPX) (incremental protocol on a cycle ergometer). Root mean square error (validity results) was 4.2 ± 3.0 mLO_2_·kg^−1^·min^−1^ for model 1, 4.1 ± 3.1 mLO_2_·kg^−1^·min^−1^ for model 2 and 3.5 ± 2.8 mLO_2_·kg^−1^·min^−1^ for model 3. Participant-independent root mean square error decreased by 15% and 18% when model 3 was compared with model 1 and 2, respectively.

Brinkløv et al. [46] developed the *InterWalk* app, integrating the InterWalk Fitness Test. The on-board accelerometer of the smartphone was used as a predictor of peak *V*O_2_ during the test. Specifically, the vector magnitude during the last 30 seconds of the test, body weight, height, and gender were used to create a linear regression equation to predict peak *V*O_2_. The criterion CRF was determined as peak *V*O_2_ assessed by CPX (graded walking test protocol on a treadmill). The overall peak VO_2_ prediction of the algorithm (*R*^2^) was 0.60 and 0.45 when the smartphone was placed in the right pocket of the pants (lower position) or jacket (upper position), respectively (*p* < 0.001). No differences were found in peak *V*O_2_ when the test was performed with or without verbal encouragement (*p* = 0.70). The reliability (intraclass correlation coefficient, ICC [95% CI]) was 0.86 [0.64–0.96] of the predicted peak VO_2_ for the lower position of the smartphone and 0.85 [0.60–0.96] for the upper position.

Brooks et al. [45] developed the *SA-6MWTapp* integrating the 6-minute walk fitness test (6MWT). They developed a distance estimation algorithm for the *SA-6MWTapp*, considering step counts from an ActiGraph GT3X and measured distance on a pre-measured 6MWT course. The best-fit algorithm was incorporated into the *SA-6MWTapp*. In addition, self-reported information from the app (age, birth date, height, and weight) and heart rate immediately at the end of the 6MWT was collected. The heart rate was taken using photoplethysmography from the user’s finger placed over the phone’s camera at the end of the test. The validation protocol was undertaken with one smartphone placed in a hip holster and the other smartphone placed in the front pants pocket. The correlation between *SA-6MWTapp* estimated distance and in-clinic measured distance along a pre-measured course was 0.88 (95% CI 0.87–0.86) and the mean difference ± SD was 7.6 ± 26 m (*p* = 0.30). The smartphone position did not influence the estimation of measured distance (*p* = 0.70). The coefficient of variation from distances estimated from the *SA-6MWTapp* was 3.2 ± 1 m (home validation phase) and highly correlated with in-clinic measured distance (*r* = 0.88 [95% CI 0.87–0.89]).

Capela et al. [47, 48] developed the *2–6MWT* app and the *TOHRC Walk Test* app, respectively. The 2-minute walk fitness test and 6MWT, respectively, were integrated into a Blackberry Z10. They used the accelerometer, gyroscope, and magnetometer of a Blackberry Z10 at approximately 50 Hz and developed an algorithm capable of estimating total distance walked, total number of steps, number of steps per length, cadence, step time (left and right steps), stride time, and step time symmetry (left and right steps). The smartphone was placed around the person’s waist using a belt that included a rear pocket (to fit the smartphone) at the center of the lower back. A digital video recorded from a separate BlackBerry 9900 smartphone was used as a gold standard. Capela et al. [48] found that the foot strike time measured with the *2–6MWT* app was within 0.07 s when compared with gold standard video recordings. Furthermore, the total distance calculated by the *2–6MWT* app was within 1 m of the measured distance. Capela et al. [47] showed that the average difference between the *TOHRC Walk Test* app and gold standard foot strike timing was 0.014 ± 0.015 s. Also, the total distance calculated by the *TOHRC Walk Test* app was within 1 m of the measured distance for all but one participant.

### App Markets Results

The app markets search led to a total of 88 apps meeting our inclusion criteria, of which 42 were stored in Google Play and 46 in App Store, with only four apps simultaneously stored on both platforms. The cost of the apps ranged from €0 to €10.99 (mean 1.24, SD 2.15) with more than half offered for free (*n* = 53, 60.22%). Google Play (*n* = 31, 73.80%) market stored more free apps than App Store (*n* = 22, 47.08%). Supplemental Tables 6–7 (in the ESM) show MARS mean and domain scores of the apps rated. Apps were sorted from highest to lowest according to the MARS mean scores (see Supplemental Tables 6–7 in the ESM). The average total MARS score was 2.97 (SD 0.73) out of 5 and 46.59% (*n* = 41) had a minimum acceptability score of 3.00. Regarding the MARS domains, functionality was the highest scoring (mean 3.85, SD 0.76), followed by aesthetics (mean 2.79, SD 1.14), information (mean 2.72, SD 0.95), engagement (mean 2.54, SD 0.86), subjective (mean 1.90, SD 1.07) and perceived impact (mean 1.70, SD 1.01). The top five ranked apps in App Store were *HRV4Training*, *MyHeart Counts*, *Fitness Test pro*, *AeroExaminer—Aerobic VO*_*2*_*Max Test and Conditioning* and *CardioCoach*, respectively. The top five ranked apps in Google Play were *HRV4Training*, *Fitness Test pro*, *iWalkAssess*, *Bruce Treadmill Test Lite*, and *Bruce Treadmill Test Protocol*, respectively.

Supplemental Tables 8–9 (in the ESM) provide the reader with a complete set of apps currently available, including a direct link (by clicking on the app’s name) to each specific app, as well as a qualitative evaluation of each of the apps. The 20-m shuttle run test was the most prevalent field-based fitness test used within Google Play and App Store apps (*n* = 31, 27.28%). Only one app (*HRV4Training*) [44] included a measure of a physiological signal and four apps calculated the distance by GPS. Sixty-one apps (69.31%) provided *V*O_2max_ estimation without considering any physiological signal, 31 (35.22%) included reference values for maximal/peak *V*O_2_ interpretation, 47 (53.40%) allowed assessments to be saved and 30 (34.09%) had the chance to add multiple users. Five apps (5.68%) required external equipment to estimate CRF, 28 (31.81%) enabled the user to export data, 31 (35.22%) enabled the user to share results in major social networks, and 49 apps of 88 (55.68%) provided test instructions to participants.

**Fig. 2:**
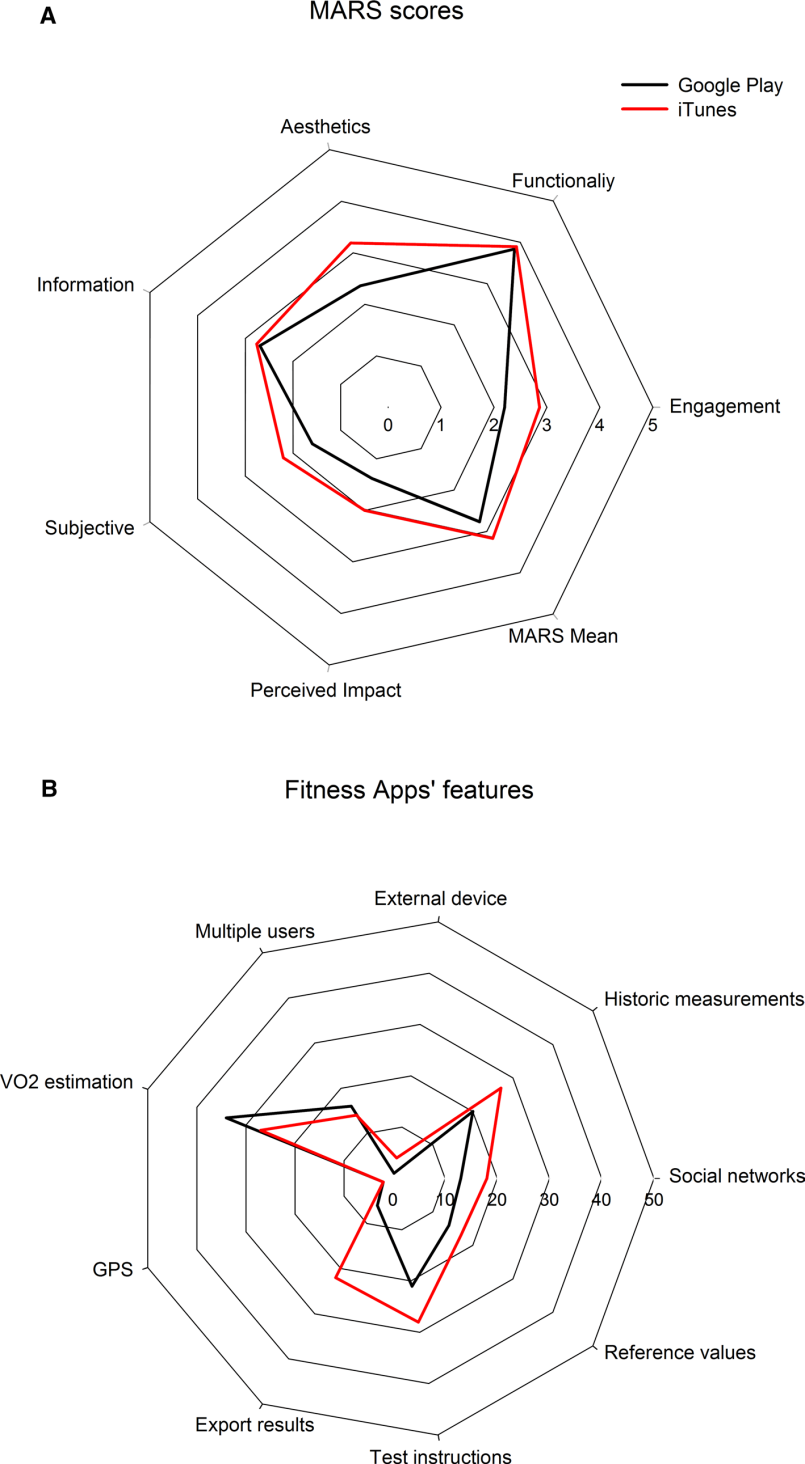
Quality scoring (*MARS*, mobile app rating scale) of the apps (**a**) and apps’ futures (**b**). In **a**, the numbers 0–5 signify the score obtained in each MARS item, whereas in **b**, the numbers 0–50 refer to the total number of apps that contain such features

Figure 2 shows a comparison between apps stored in Google Play and App Store regarding the quality scoring with MARS (A) and the apps’ features (B). MARS mean was slightly higher for App Store apps in comparison with Google Play (3.17 vs. 2.77). Likewise, all the MARS domains obtained higher scores for App Store apps. Regarding the apps’ features, the App Store repository stored more apps than Google Play in all items except for the *V*O_2_ estimation item. A positive association was observed between the cost of apps and the total MARS mean score (*r *= 0.46; *p *< 0.001). The total number of features was positively associated with the total MARS mean score (*r *= 0.55; *p *< 0.001).

## Discussion

The purpose of this report was three-fold: first, to systematically review the validity and reliability of CRF apps assessment available in the scientific literature and app markets; second, to provide evidence-based practical recommendations; and third, to stimulate a scientific discussion on how the information retrieved from these apps might have clinical relevance for the assessment of CV health, as well as for sports performance and training.

One of our major findings is that, despite having identified 88 fitness apps, only five have been tested scientifically; all five for validity [44–48] and only two for reliability [45, 46]. Three of the five apps were scored as having a moderate to good validity [44–46]. Nevertheless, there were some limitations, for example, (1) none of the five apps were stored on both app markets for free download and two were not for public use; (2) the sample size of the validation studies was small, and (3) four studies [45–48] used algorithms designed from data collected by the smartphone’s accelerometer, which could impact applicability to other smartphones since the algorithms may not provide valid data when used with other smartphone models.

**Fig. 3:**
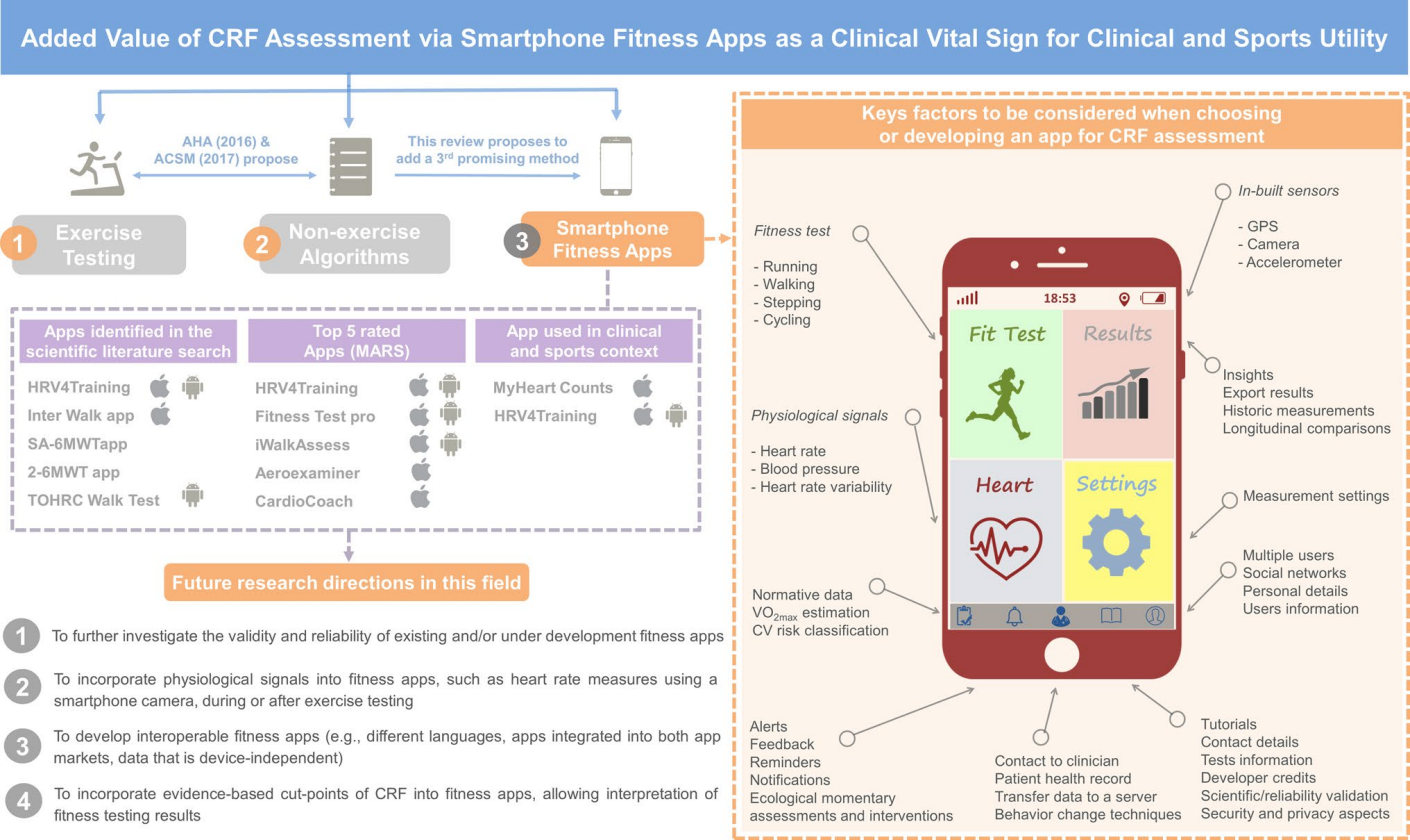
Apps identified in the scientific literature search and in the app markets review, future research directions, and key factors to be considered when selecting or developing an app for assessing cardiorespiratory fitness. *CRF* cardiorespiratory fitness, *CVD* cardiovascular disease, *MARS* Mobile App Rating Scale

Despite these limitations, indicating there is not the ability to reach a consensus on a preferred fitness app, some may be useful, particularly compared with performing no CRF assessment. Among them, the *MyHeart Count* app currently possesses the greatest clinical utility among the commercial apps reviewed. The *MyHeart Count* estimates CRF by means of the 6MWT, with more than 400 customer ratings and a current score of 4.5 out of 5 (see Supplemental Tables 8–9 in the ESM). In addition, its feasibility has been published [26] and the app contains many of the conditions described in Fig. 3. Notwithstanding, the main limitation is that its validity and reliability have not been tested to date; therefore, caution should be taken with its use. Furthermore, *MyHeart Counts* is currently available in the United States alone, and only to iPhone users (version 5S and later). Regarding the sports field, for both recreational and high-performance sports purposes, the *HRV4training* app currently has proven to be the most useful tool. However, coaches and athletes should exercise caution when using this app. Although its validity for determining CRF is good, no reliability data is currently available. Moreover, CRF estimation is exclusively available for runners and cyclists linking the *HV4training* app to the *Strava* app and using a heart rate monitor and a power meter during their workouts. Also, the *HV4training* app is the most expensive within the two main markets analyzed in this review.

### Evidence-Based Practical Recommendations

In this review, we sought to contribute to this developing field of research by identifying limitations of current apps and outlining the desirable characteristics that are generalizable to multiple populations with differing needs. Accordingly, we discuss some fundamental points that should be considered when selecting an app among the many options available or when developing a new app. Future research directions based on the knowledge gaps identified herein are also considered.

Most of the apps assessed were based on the maximal CRF test, which, for patient populations, is a clinical standard requiring professionals with specialized training. To broaden applicability, validation of apps using sub-maximal tests to estimate CRF (e.g., 6-minute walk test, 1-mile walk test, etc.) enhance the ability to remotely assess CRF in safer and more feasible conditions. In this regard, a recent major study demonstrated the impact of changes in submaximal CRF on health outcomes [49]. Additionally, we have found that most apps served as a simple CRF calculator while only one included a physiological signal (e.g., heart rate and heart rate variability) to estimate CRF. The integration of physiological signals into apps might provide more accurate data to better estimate an individual’s CVD risk and/or sports performance. Therefore, technology advancements that incorporate relevant physiological signals into apps is another venue for future research in this area. According to our review, the modes of exercise used for CRF testing include running, walking, stepping, and cycling, with running tests as the most prevalent mode. However, care should be taken when this testing modality is used in individuals with physical limitations and when testing takes place remotely.

Another important aspect to be considered when choosing an app is where the fitness testing will take place. In this context, apps including step tests are a feasible solution when testing is performed in a room with limited resources. Otherwise, the external equipment needed, such as a treadmill or stationary bicycle, is another key factor when selecting an app. Those apps that require additional resources and equipment will make large-scale assessments more challenging and less feasible. Therefore, whenever possible, CRF tests without the need for additional equipment are preferable. It is important to note that submaximal exercise tests with fewer resource and equipment requirements can provide valuable information although they are not as precise as maximal exercise testing [37].

The cost and language of apps are also important factors, making apps universally accessible to individuals across broad socioeconomic strata. Most apps examined are available in English and half of them are offered at no cost. Another major problem identified in this systematic review was that very few apps (i.e., only four out of 88 [4.54%]) were simultaneously available on both the Google Play and App Store platforms. The low number of apps that are on both platforms can be attributed to different reasons. For instance, apps in the App Store must meet a quality guideline review prior to publication, demanding higher app quality than Google Play; however, the publishing cost is higher than with Google Play. This fact is likely the cause of the higher cost of apps in the App Store. Thus, app developers may choose to publish apps in one or another market depending on these factors. This is an important limitation since, ideally, a researcher, clinician or sports professional would like to use the same app regardless of which type of smartphone the individual may have. Ideally, future efforts in the field should be focused on published apps in the most spoken languages worldwide, that are low-cost, and stored in both app markets for optimal clinical and sports application.

An additional drawback of the scientific and commercial apps reviewed herein is the lack of data on interoperability. Despite the information collected from apps, opportunities for connected healthcare with respect to CRF assessment remains suboptimal; the transmission of patient-generated data, stored in Android and Apple devices, to the patients’ electronic health records has been previously documented [50, 51] and should be considered for CRF assessment. In order to achieve app interoperability, a plan is needed to support developments in privacy and data security, as well as interoperability across smartphone devices and app markets, extending data from devices to electronic health records [52]. As mHealth matures, health information technology interoperability will bring a real integration of patient-generated CRF data into electronic health records, making data device-independent.

In addition, most of the apps already use existing and scientifically validated CRF field-based fitness tests (e.g., 20-m shuttle run test, 6-minute walk test, Cooper Test) transformed into an app format. However, the ability of the resulting apps to assess CRF against a criterion method has rarely been tested. Thus, it is highly recommended, whenever possible, to select scientifically validated apps, and for researchers to test the validity and reliability of existing and newly developed apps. Likewise, other functionalities such as the inclusion of test instructions, the capability to store repeated measurements to later perform longitudinal comparisons, the possibility to export data entered and the main results of the test, the integration of multiple users, and the ability to generate feedback based on results are important factors that should be kept in mind when selecting an app or developing a new one.

### Future Potential for Fitness Apps in a Clinical and Sports Context

Figure 3 presents the main characteristics that would emulate a high-quality fitness app for clinical and sports fields. This information will assist researchers to work together with app developers to design better apps in the future.

In this sense, future potential of the use of apps for CRF testing in a clinical context might encourage patients to seek knowledge about their CRF level, which would, in turn, be translated into the management risk of CVDs [53]. In addition, high-quality fitness apps would be relatively inexpensive whereas the assessment of well established risk factors for developing a CVD (e.g., cholesterol and blood pressure) requires equipment with a high economic cost. Furthermore, monitoring is usually reserved for individuals with increased risk for or established CVD. Thanks to the universality of apps, people of all ages and socioeconomic status might be encouraged to self-assess their CRF to estimate the lifetime risk of CVD. Low CRF is independent of other CVD risk conditions traditionally controlled in the clinics (e.g., obesity, hypertension, type 2 diabetes, dyslipidemia), therefore the integration of CRF assessments through apps would enhance the traditional method for estimating CVD risk into clinical workflows. Patients do not always recognize themselves as being at CVD risk, hence CRF apps with alarms in case of low CRF would favor the early detection of CV abnormalities.

In the sports context, future apps for CRF testing might allow coaches to integrate this measurement into their routine practice, overcoming the limitations of traditional methods noted above. For instance, a desirable fitness app might have two unique components, one for the athlete to track measurements and the other for coaches to manage data collected from the individual or team as a whole. In this context, coaches might receive athletes’ data remotely, making it more feasible to make adjustments in training programs on a daily basis. Along the same lines, these fitness apps would incorporate a training index, based on daily CRF measurement and other acute stressors, to predict trainability of athletes according to current physiological status. The capability of fitness apps to collect and store CRF measurements effortlessly would make the interpretation of acute and chronic training loads more feasible. Although CRF is *per se* a recognized indicator of sports performance for recreational and elite athletes, the future ability of fitness apps to collect other physiological and non-physiological parameters may enrich the interpretation of CRF measurements in this field.

## Limitations and Strengths

The main limitation of this review was the small number of scientific studies identified. A second important limitation was the bias found in some validation manuscripts, which makes it difficult to draw robust conclusions. Further, even though we provided a list of apps currently available in app markets, it is important to note that the volume and turnover of apps are high; thus, it is likely that new applications will appear while others assessed in the current analysis will be defunct in the near future. The strengths of our review include a comprehensive analysis and discussion concerning the opportunities that apps provide for the objective and remote assessment of CRF and their usefulness for clinical and sports/training purposes [1–6, 8–10]. Specifically, our review contributes to the field by providing (1) information on the validity and reliability of apps currently available in the scientific literature; (2) a comprehensive list of apps currently available in app markets, including a qualitative rating of each in order to assist readers with selection of the best apps (Supplemental Tables 6–9 in the ESM, which include a direct link to each app); (3) a list of the key characteristics that a fitness app should have in order to assist readers with the selection of apps, as well as app developers to design better apps in the future; and (4) a list of recommendations for future research directions based on knowledge gaps identified during this systematic review.

## Conclusions

There is no doubt that we are witnessing the beginning of a new technologic era in healthcare and sports; however, the validity/reliability of the CRF assessment should be improved in a manner consistent with technological development. In fact, the results from this review demonstrate that few presently available apps have been empirically evaluated and among those that have, not all are valid or reliable. In addition, commercially available apps are mostly of low-to-moderate quality, suggesting that the potential of apps for assessing CRF has yet to be realized. Lastly, this manuscript has identified evidence-based practical recommendations for the future development of apps that objectively and remotely assess CRF as a complementary tool to traditional methods for estimating lifetime CVD risk and for improving athletes’ performance. Likewise, sports practitioners will be able to take advantage of the opportunities that fitness apps offer to evaluate the CRF level of clients remotely and to monitor fitness changes. Collectively, we believe that expanding digitalization is a key component of the future of healthcare and sports, and in turn capitalizing on digitalization for the refinement of CRF assessment, now considered a vital sign [37], is an important objective that requires continued inquiry.

## Electronic supplementary material

Below is the link to the electronic supplementary material.

Supplementary material 1 (PDF 815 kb)
