# Supplementary material for: A Systematic Review of Fitness Apps and Their Potential Clinical and Sports Utility for Objective and Remote Assessment of Cardiorespiratory Fitness
Source: Sports Med. 2019 Mar 1;49(4):587–600. doi: 10.1007/s40279-019-01084-y (PMC6422959; doi:10.1007/s40279-019-01084-y)
Supplement: Supplementary file 1 — Supplementary material 1 (PDF 815 kb) [file 40279_2019_1084_MOESM1_ESM.pdf]

**ELECTRONIC SUPPLEMENTARY MATERIAL** from the article by Muntaner-Mas et al. "A Systematic Review of Fitness Apps and Their Potential Clinical and Sports Utility for Objective and Remote Assessment of Cardiorespiratory Fitness"


**Table 1.** Scientific literature search strategy used and number of articles found in Pubmed. ............................................ **2**

**Table 2**. Scientific literature search strategy used and number of articles found in Web of Science® .............................. **3**

**Table 3**. Scientific literature search strategy used and number of articles found in Scopus™.......................................... **4**

**Table 4**. Scientific literature search strategy used and number of articles found in SPORTDiscus. ............................. ….**5**

**Table 5**. Apps' markets search strategy used and a number of apps in the iTunes and Google Play stores. ....................... **6**

**Table 6**. Mobile App Rating Scale (MARS) mean and domains scores of fitness apps stored in App Store (iTunes). .... **7-8**

**Table 7.** Mobile App Rating Scale (MARS) mean and domains scores of fitness apps stored in Google Play apps...... **9-10**

**Table 8.** Qualitative assessment of fitness apps stored in App Store (iTunes)............................................................ **11-14**

**Table 9.** Qualitative assessment of fitness apps stored in Google Play....................................................................... **15-18**

**ELECTRONIC SUPPLEMENTARY MATERIAL** from the article by Muntaner-Mas et al. "A Systematic Review of Fitness Apps and Their Potential Clinical and Sports Utility for Objective and Remote Assessment of Cardiorespiratory Fitness"

**Table 1.** Scientific literature search strategy used and number of articles found in Pubmed.

| **Search criteria 1** | **Search criteria 2** | |
|---|---|---|
| | **2.1 Assessment terms** | **2.2 Cardiorespiratory fitness** |
| Mobile Applications (Mesh) OR Cell Phones (Mesh) OR Smartphone (Mesh) OR Smartphone App OR Smartphone Application OR iPhone App OR Android App | Exercise Tolerance (Mesh) OR Exercise Test (Mesh) OR Physical Fitness (Mesh) OR Athletic Performance (Mesh) OR Reproducibility Of Results (Mesh) OR Task Performance And Analysis (Mesh) | Cardiorespiratory Fitness (Mesh) OR Oxygen Consumption OR (Mesh) Walk Test (Mesh) OR Walking Speed (Mesh) OR Cardiovascular Fitness OR Aerobic Fitness OR Aerobic Capacity OR Maximal Oxygen Consumption OR $VO_2$max |
| **Items found (Combining Criteria (1 "AND" 2.1) OR (1 "AND" 2.1))** | 561 | 35 |
| **Total items found (after deleting duplicates)** | **575** | |

**General search equation (n = 575):**

(((((((((((("Mobile Applications"[Mesh]) OR "Cell Phones"[Mesh]) OR "Smartphone"[Mesh])) OR smartphone app) OR smartphone application) OR iPhone app) OR Android App)) AND (((((("Exercise Tolerance"[Mesh]) OR "Exercise Test"[Mesh]) OR "Physical Fitness"[Mesh]) OR "Athletic Performance"[Mesh]) OR "Reproducibility of Results"[Mesh]) OR "Task Performance and Analysis"[Mesh]) AND ("2000/01/01"[PDat]: "2018/09/30"[PDat]))))) OR (((((((((((("Mobile Applications"[Mesh]) OR "Cell Phones"[Mesh]) OR "Smartphone"[Mesh])) OR smartphone app) OR smartphone application) OR iPhone app) OR Android App)) AND ((((((((("Cardiorespiratory Fitness"[Mesh]) OR "Oxygen Consumption"[Mesh]) OR "Walk Test"[Mesh]) OR "Walking Speed"[Mesh])) OR cardiovascular fitness) OR aerobic fitness) OR aerobic capacity) OR maximal oxygen consumption) OR VO2max) AND ("2000/01/01"[PDat] : "2018/09/30"[PDat]))))

**Table 2**. Scientific literature search strategy used and number of articles found in Web of Science®.

| **Search criteria 1** | **Search criteria 2** | |
|---|---|---|
| | **2.1 Assessment terms** | **2.2 Cardiorespiratory fitness** |
| Mobile Applications OR Smartphone OR Smartphone App OR Smartphone Application OR iPhone App OR Android App | Physical Fitness OR Physical Condition | Cardiorespiratory Fitness OR Oxygen Consumption OR Cardiovascular Fitness OR Aerobic Fitness OR Aerobic Capacity OR Maximal Oxygen Consumption OR $VO_2$max |
| **Items found (Combining Criteria (1 "AND" 2.1) OR (1 "AND" 2.1))** | 771 | 79 |
| **Total items found (after deleting duplicates)** | **804** | |

**General search equation (n = 804):**

(TS=(mobile applications) OR TS=(smartphone) OR TS=(smartphone app) OR TS=(smartphone application) OR TS=(IPhone App) OR TS=(Android App)) AND (TS=(physical fitness) OR TS=(physical condition)) OR (TS=(mobile applications) OR TS=(smartphone) OR TS=(smartphone app) OR TS=(smartphone application) OR TS=(IPhone App) OR TS=(Android App)) AND (TS=(cardiorespiratory fitness) OR TS=(oxygen consumption) OR TS=(cardiovascular fitness) OR TS=(aerobic fitness) OR TS=(aerobic capacity) OR TS=(maximal oxygen consumption) OR TS=(VO2max))

**Table 3**. Scientific literature search strategy used and number of articles found in Scopus™.

| Search criteria 1 | Search criteria 2 | |
|---|---|---|
| | **2.1 Assessment terms** | **2.2 Cardiorespiratory fitness** |
| Mobile Applications OR Smartphone OR Smartphone App OR Smartphone Application OR iPhone App OR Android App | Physical Fitness OR Exercise Test | Cardiorespiratory Fitness OR Oxygen Consumption OR Cardiovascular Fitness OR Aerobic Fitness OR Aerobic Capacity |
| **Items found (Combining Criteria (1 “AND” 2.1) OR (1 “AND” 2.1))** | 528 | 428 |
| **Total items found (after deleting duplicates)** | **826** | |

**General search equation (n = 826):**

(ALL ( "mobile applications" ) OR ALL ( "smartphone" ) OR ALL ( "smartphone app" ) OR ALL ( "smartphone application" ) OR ALL ( "iPhone App" ) OR ALL ( "Android App" ) AND ( ALL ( "Physical Fitness" ) OR ALL ( "Exercise test" ) ) AND PUBYEAR > 2000) OR ((ALL ( "mobile applications" ) OR ALL ( "smartphone" ) OR ALL ( "smartphone app" ) OR ALL ( "smartphone application" ) OR ALL ( "iPhone App" ) OR ALL ( "Android App" )) AND (ALL ( "Cardiorespiratory Fitness" ) OR ALL ( "Cardiovascular Fitness" ) OR ALL ( "Aerobic Fitness" ) OR ALL ( "Oxygen Consumption" ) OR ALL ( "Aerobic Capacity" )) AND PUBYEAR > 2000)

**ELECTRONIC SUPPLEMENTARY MATERIAL** from the article by Muntaner-Mas et al. "A Systematic Review of Fitness Apps and Their Potential Clinical and Sports Utility for Objective and Remote Assessment of Cardiorespiratory Fitness"

**Table 4**. Scientific literature search strategy used and number of articles found in SPORTDiscus.

| Search criteria 1 | Search criteria 2 | |
|---|---|---|
| | **2.1 Assessment terms** | **2.2 Cardiorespiratory fitness** |
| Mobile Applications OR Smartphone OR Smartphone App OR Smartphone Application OR iPhone App OR Android App | Physical Fitness OR Exercise Tests OR Reproducibility Of Results | Cardiorespiratory Fitness OR Oxygen Consumption OR Cardiovascular Fitness OR Aerobic Fitness OR Aerobic Capacity OR Maximal Oxygen Consumption OR VO2max |
| **Items found (Combining Criteria 1 "AND" 2)** | 523 | 187 |
| **Total items found (after deleting duplicates)** | **591** | |

**General search equation (n = 591):**

( TX ( mobile applications OR smartphone OR smartphone app OR smartphone application OR iPhone App OR Android App) AND TX (physical fitness OR exercise tests OR Reproducibility Of Results ) ) OR ( TX ( mobile applications OR smartphone OR smartphone app OR smartphone application OR iPhone App OR Android App ) AND TX ( Cardiorespiratory Fitness OR Oxygen Consumption OR Cardiovascular Fitness OR Aerobic Fitness OR Aerobic Capacity OR Maximal Oxygen Consumption OR VO2max ) )

**Table 5**. Apps’ markets search strategy used and a number of apps in the iTunes and Google Play stores.

| **Limits** | **Search criteria** | |
|---|---|---|
| | **1.1 Assessment terms** | **1.2 Cardiorespiratory fitness** |
| Smartphone applications the focus in cardiorespiratory fitness testing | Fitness test | Walk test OR VO2max OR Yo-Yo test |
| **Items found** | 97 | 595 |
| **Total items found** | **692** | |

**App Store (iTunes)**

Dates of the search: October 2018 Total #Hits = 201

#Hits by search terms:
Fitness test [97], walk test [14], VO2max [33], yo-yo test [58]

**Google Play**

Dates of the search: October 2018 Total #Hits = 490

#Hits by search terms:
Walk test [249], VO2max [241]

**Table 6.** Mobile App Rating Scale (MARS) mean and domains scores of fitness apps stored in App Store (iTunes).

| | App name | Engagement | Functionality | Aesthetics | Information | MARS Mean | Subjective | Perceived Impact |
|---|---|---|---|---|---|---|---|---|
| 1 | HRV4Training | 5.00 | 5.00 | 5.00 | 5.00 | 5.00 | 5.00 | 5.00 |
| 2 | MyHeart Counts | 4.20 | 5.00 | 5.00 | 3.86 | 4.51 | 4.50 | 4.50 |
| 3 | Fitness Test pro | 3.60 | 5.00 | 5.00 | 3.83 | 4.36 | 4.50 | 4.17 |
| 4 | AeroExaminer - Aerobic VO2 Max Test & Conditioning | 4.40 | 3.75 | 4.33 | 4.57 | 4.26 | 4.50 | 2.83 |
| 5 | CardioCoach | 4.00 | 3.75 | 5.00 | 3.86 | 4.15 | 4.00 | 4.00 |
| 6 | iWalkAssess | 2.40 | 4.75 | 4.00 | 4.67 | 3.95 | 3.25 | 2.50 |
| 7 | Team Bleep Test | 3.80 | 4.25 | 4.33 | 3.29 | 3.92 | 3.25 | 3.83 |
| 8 | CycleCoach - Fitness Test | 3.60 | 4.00 | 4.33 | 3.20 | 3.78 | 4.25 | 2.67 |
| 9 | Multi Stage Pacer Test | 2.80 | 4.75 | 4.00 | 3.25 | 3.70 | 2.00 | 1.17 |
| 10 | Police Fitness | 2.80 | 4.75 | 4.00 | 3.17 | 3.68 | 1.75 | 1.00 |
| 11 | Bleep Test Pro - 15m & 20m - Beep Test Shuttle Run | 2.60 | 5.00 | 4.33 | 2.75 | 3.67 | 1.00 | 1.50 |
| 12 | BEEP TEST TRAINER | 3.40 | 4.25 | 4.00 | 3.00 | 3.66 | 3.25 | 2.67 |
| 13 | Max O2 | 3.00 | 4.00 | 4.33 | 3.00 | 3.58 | 1.00 | 1.33 |
| 14 | examen de aptitud | 2.40 | 4.50 | 3.67 | 3.50 | 3.52 | 2.00 | 2.50 |
| 15 | Multi Stage VO2 max Beep Test | 4.00 | 4.00 | 3.67 | 2.33 | 3.50 | 4.00 | 2.33 |
| 16 | Ekblom-Bak Cycle Test | 2.80 | 4.00 | 2.33 | 4.60 | 3.43 | 1.25 | 2.00 |
| 17 | AF Fitness Assessment | 2.40 | 4.00 | 2.33 | 4.83 | 3.39 | 1.75 | 2.67 |
| 18 | BEEP TEST TEAM TRAINER | 2.80 | 4.00 | 4.33 | 2.40 | 3.38 | 1.50 | 2.00 |
| 19 | Bleep Test - Fitness Tests | 3.40 | 3.75 | 4.00 | 2.00 | 3.29 | 1.75 | 2.33 |
| 20 | 3 Minute Step Test - DIY Fitness Assessment | 2.80 | 4.50 | 3.33 | 2.50 | 3.28 | 2.00 | 2.50 |
| 21 | Bleep.Test | 2.80 | 4.25 | 2.67 | 3.00 | 3.18 | 2.00 | 1.33 |
| 22 | Beep Test Training Guide | 2.40 | 4.00 | 2.00 | 4.29 | 3.17 | 1.75 | 4.17 |
| 23 | Treadmill Tracker | 3.00 | 3.50 | 2.33 | 3.75 | 3.15 | 2.25 | 2.00 |
| 24 | Beep Fitness Test | 2.80 | 4.00 | 3.67 | 2.00 | 3.12 | 2.25 | 1.00 |
| 25 | UFT - Uber Fitness Test | 2.80 | 4.50 | 2.67 | 2.50 | 3.12 | 2.00 | 1.00 |
| 26 | The Beep Test | 2.60 | 3.50 | 3.67 | 2.57 | 3.08 | 2.50 | 2.83 |
| 27 | FitnessMeter - Test & Measure | 2.80 | 3.00 | 2.33 | 4.17 | 3.08 | 3.25 | 2.33 |

| | | | | | | | | |
|---|---|---|---|---|---|---|---|---|
| **28** | 6MWT Lite | 3.80 | 2.50 | 3.33 | 2.50 | 3.03 | 1.50 | 1.50 |
| **29** | 6MWT | 3.60 | 2.50 | 3.00 | 2.50 | 2.90 | 1.00 | 1.50 |
| **30** | 6MWD | 2.40 | 4.50 | 2.67 | 2.00 | 2.89 | 1.00 | 1.00 |
| **31** | The Beep Test Free | 2.60 | 3.75 | 2.00 | 3.00 | 2.84 | 2.75 | 1.67 |
| **32** | Fit Test | 3.40 | 4.25 | 2.67 | 1.00 | 2.83 | 2.50 | 1.00 |
| **33** | Beep Test | 2.20 | 4.00 | 3.33 | 1.67 | 2.80 | 2.00 | 1.00 |
| **34** | Bleep Test Lite | 2.60 | 3.75 | 3.33 | 1.50 | 2.80 | 1.75 | 1.00 |
| **35** | Bleep Test Solo | 2.60 | 3.75 | 3.33 | 1.50 | 2.80 | 1.75 | 1.00 |
| **36** | T4T - Test4Training | 2.00 | 4.00 | 3.00 | 2.00 | 2.75 | 1.00 | 1.00 |
| **37** | Prueba Pacer VO2 Max Cardio y Shuttle Bleep | 2.60 | 3.75 | 2.33 | 2.00 | 2.67 | 2.75 | 2.33 |
| **38** | VO2 Prueba De Pitidos Max - Pacer, Bleep & Shuttle | 3.20 | 3.25 | 2.00 | 1.67 | 2.53 | 1.00 | 1.67 |
| **39** | BEEP TEST SLIM | 1.40 | 3.25 | 2.00 | 2.80 | 2.36 | 1.50 | 1.17 |
| **40** | Music Beep Test - Pacer & Shuttle Run Fitness Test | 2.20 | 4.00 | 2.00 | 1.00 | 2.30 | 1.75 | 1.50 |
| **41** | Shuttle Fit Trainer | 2.00 | 3.25 | 2.33 | 1.50 | 2.27 | 1.25 | 1.00 |
| **42** | Test de Cooper | 2.40 | 3.25 | 1.33 | 2.00 | 2.25 | 1.00 | 1.50 |
| **43** | YO YO Endurance Test | 2.20 | 2.00 | 1.33 | 3.25 | 2.20 | 1.00 | 1.00 |
| **44** | Beep Test: Examen de Ejército Policía Militares | 1.80 | 3.50 | 2.33 | 1.00 | 2.16 | 1.00 | 1.00 |
| **45** | Beep Test (Multi-Stage) | 1.80 | 3.75 | 1.33 | 1.00 | 1.97 | 1.00 | 1.00 |
| **46** | VO2max Calculator | 1.20 | 2.00 | 2.33 | 1.00 | 1.63 | 1.00 | 1.00 |
| | **Average (SD)** | **2.86 (0.77)** | **3.89 (0.71)** | **3.19 (1.04)** | **2.76 (1.11)** | **3.17 (0.70)** | **2.20 (1.15)** | **2.00 (1.08)** |

**ELECTRONIC SUPPLEMENTARY MATERIAL** from the article by Muntaner-Mas et al. "A Systematic Review of Fitness Apps and Their Potential Clinical and Sports Utility for Objective and Remote Assessment of Cardiorespiratory Fitness"

**Table 7.** Mobile App Rating Scale (MARS) mean and domains scores of fitness apps stored in Google Play apps.

| | App name | Engagement | Functionality | Aesthetics | Information | MARS Mean | Subjective | Perceived Impact |
|---|---|---|---|---|---|---|---|---|
| **1** | HRV4Training | 5.00 | 5.00 | 5.00 | 5.00 | 5.00 | 5.00 | 5.00 |
| **2** | Fitness Test pro | 3.60 | 5.00 | 5.00 | 3.83 | 4.36 | 4.50 | 4.17 |
| **3** | iWalkAssess | 2.40 | 4.75 | 4.00 | 4.67 | 3.95 | 3.25 | 2.50 |
| **4** | Bruce Treadmill Test Lite | 2.60 | 5.00 | 3.33 | 4.00 | 3.73 | 1.75 | 1.83 |
| **5** | Bruce Treadmill Test Protocol | 2.80 | 4.50 | 3.33 | 3.20 | 3.46 | 1.50 | 1.50 |
| **6** | Physical V02 Fitness Beep Test | 3.00 | 4.25 | 3.33 | 3.20 | 3.45 | 2.25 | 1.50 |
| **7** | Marine Corps PT Calculator - USMC Physical Fitness | 2.80 | 5.00 | 3.33 | 2.60 | 3.43 | 1.50 | 1.33 |
| **8** | TOHRC Walk Test | 3.00 | 4.25 | 3.33 | 3.14 | 3.43 | 2.25 | 1.00 |
| **9** | 3 Minute Step Test | 2.80 | 4.75 | 2.33 | 3.60 | 3.37 | 2.50 | 1.67 |
| **10** | blp - The Bleep Test App | 2.40 | 4.25 | 3.00 | 3.20 | 3.21 | 1.50 | 1.17 |
| **11** | Functional Assessment + G-Code | 2.60 | 3.75 | 3.33 | 3.00 | 3.17 | 2.00 | 1.50 |
| **12** | Run U2 | 3.00 | 4.00 | 2.67 | 2.50 | 3.04 | 2.00 | 1.00 |
| **13** | Fitness Assessments | 2.40 | 4.25 | 2.67 | 2.75 | 3.02 | 1.75 | 1.50 |
| **14** | Test de Léger | 1.80 | 3.75 | 3.00 | 3.00 | 2.89 | 1.25 | 1.00 |
| **15** | 12 Minute Vo2 Max Run Test | 2.80 | 3.25 | 3.33 | 2.00 | 2.85 | 1.25 | 1.17 |
| **16** | Physical Fitness V02 Beep Test | 2.80 | 3.25 | 3.33 | 2.00 | 2.85 | 1.25 | 1.17 |
| **17** | Shuttle Run VO2Max Pacer Test | 2.80 | 3.25 | 3.33 | 2.00 | 2.85 | 1.25 | 1.17 |
| **18** | Calculadora Funcional Trainer | 2.20 | 4.00 | 2.67 | 2.50 | 2.84 | 1.00 | 1.00 |
| **19** | Beep Test Pro | 2.60 | 4.25 | 1.67 | 2.75 | 2.82 | 2.50 | 1.00 |
| **20** | Yo-Yo Intermittent Pro | 3.00 | 3.75 | 2.00 | 2.40 | 2.79 | 2.00 | 1.00 |
| **21** | Test de marcha de 6 minutos | 2.60 | 2.75 | 3.33 | 2.25 | 2.73 | 1.25 | 1.00 |
| **22** | Beep Test Calculator | 1.80 | 5.00 | 2.00 | 2.00 | 2.70 | 1.25 | 1.00 |
| **23** | Bruce Protocol Stress Calci | 1.80 | 5.00 | 2.00 | 2.00 | 2.70 | 1.25 | 1.00 |
| **24** | Army Fitness Calculator - APFT | 1.80 | 3.75 | 2.67 | 2.50 | 2.68 | 1.00 | 1.00 |
| **25** | Cardio Exercise Prescription | 2.20 | 4.25 | 2.00 | 2.00 | 2.61 | 1.00 | 1.50 |
| **26** | Astrand Cycle Fitness Calci | 1.00 | 4.50 | 2.33 | 2.50 | 2.58 | 1.00 | 1.00 |
| **27** | VO2 Max Calculator | 1.00 | 4.50 | 2.33 | 2.50 | 2.58 | 1.00 | 1.00 |

| | | | | | | | | |
|---|---|---|---|---|---|---|---|---|
| **28** | FitCal - Fitness Calculators | 2.40 | 3.50 | 1.33 | 2.80 | 2.51 | 1.25 | 2.50 |
| **29** | Test VMA Pro | 2.80 | 2.50 | 2.00 | 2.60 | 2.48 | 2.00 | 1.17 |
| **30** | V02 Calculadora | 1.80 | 3.50 | 1.67 | 2.80 | 2.44 | 1.00 | 1.00 |
| **31** | eRunners | 2.40 | 3.25 | 1.00 | 2.80 | 2.36 | 1.50 | 1.50 |
| **32** | Prueba Beep | 1.80 | 3.00 | 1.67 | 2.00 | 2.12 | 1.00 | 1.00 |
| **33** | Yo-Yo Test Intermitente | 1.80 | 3.00 | 1.67 | 2.00 | 2.12 | 1.00 | 1.00 |
| **34** | AG-Evaluapp - Fitness Tests | 1.40 | 4.25 | 1.00 | 1.60 | 2.06 | 1.00 | 1.00 |
| **35** | FitCalc+ Fitness & Health Calculator - Gym Tools | 1.20 | 4.25 | 1.00 | 1.67 | 2.03 | 1.00 | 1.00 |
| **36** | 2.4 Km Run Test (Fitness Test) | 1.20 | 3.00 | 1.00 | 2.60 | 1.95 | 1.00 | 1.00 |
| **37** | Beep Test vocacional Ejército | 1.20 | 3.50 | 1.00 | 2.00 | 1.93 | 1.00 | 1.00 |
| **38** | Fitga Fitness Tracker | 1.40 | 2.50 | 1.00 | 2.60 | 1.88 | 1.00 | 1.00 |
| **39** | Fitness Test | 1.40 | 2.25 | 1.00 | 2.75 | 1.85 | 1.00 | 1.00 |
| **40** | 12 Minute Run Test (Cooper Test) | 1.00 | 2.75 | 1.00 | 2.60 | 1.84 | 1.00 | 1.00 |
| **41** | Astrand Treadmill Test (Vo2max Test) | 1.00 | 2.75 | 1.00 | 2.60 | 1.84 | 1.00 | 1.00 |
| **42** | Balke Vo2max Test | 1.00 | 2.75 | 1.00 | 2.60 | 1.84 | 1.00 | 1.00 |
| | **Average** | **2.20 (0.83)** | **3.83 (0.80)** | **2.36 (1.08)** | **2.69 (0.71)** | **2.77 (0.70)** | **1.59 (0.89)** | **1.38 (0.81)** |

**ELECTRONIC SUPPLEMENTARY MATERIAL** from the article by Muntaner-Mas et al. “A Systematic Review of Fitness Apps and Their Potential Clinical and Sports Utility for Objective and Remote Assessment of Cardiorespiratory Fitness”

**Table 8.** Qualitative assessment of fitness apps stored in App Store (iTunes).

| | App name | Score | Ratings | Price | Test mode | Test instructions | HR | GPS | VO2 estimation | External devices needed |
|---|---|---|---|---|---|---|---|---|---|---|
| 1 | HRV4Training | 4.2 | 13 | 10.99 € | Published algorithms | Yes | Yes | - | Yes | - |
| 2 | MyHeart Counts | 4.5 | 132 | 0.00 € | 6 minutes walk test | Yes | - | Yes | Yes | - |
| 3 | Fitness Test pro | 0 | 0 | 3.49 € | Coopers 12-min run, Bruce treadmill test, Any distance run test, Beep-test | Yes | - | - | Yes | - |
| 4 | AeroExaminer - Aerobic VO2 Max Test & | 0 | 0 | 4.49 € | beep test, Yo-Yo Intermittent and a Treadmill Tests | Yes | - | - | Yes | Yes (treadmill) |
| 5 | CardioCoach | 0 | 0 | 0.00 € | - | - | - | - | - | - |
| 6 | iWalkAssess | 0 | 0 | 0.00 € | 10-metre walk test; 6-minute walk test | Yes | - | - | - | - |
| 7 | Team Bleep Test | 0 | 0 | 1.09 € | YO YO Endurance Test, Standard Beep Test, Uk police test, UK Fire | - | - | - | - | - |
| 8 | CycleCoach - Fitness Test | 0 | 0 | 0.00 € | Cycling test | Yes | - | - | - | Yes (bike) |
| 9 | Multi Stage Pacer Test | 0 | 0 | 0.00 € | 20 meter shuttle run test | - | - | - | Yes | - |
| 10 | Police Fitness | 0 | 0 | 2.29 € | Chester test & 20 meter shuttle run test | Yes | - | - | - | - |
| 11 | Bleep Test Pro - 15m & 20m - Beep Test Shuttle Run | 0 | 0 | 1.09 € | 15 & 20 meter shuttle run test | - | - | - | - | - |
| 12 | BEEP TEST TRAINER | 0 | 0 | 3.49 € | 20 meter shuttle run test | Yes | - | - | Yes | - |
| 13 | Max O2 | 0 | 0 | 0.00 € | Ebbeling 1, Balke 1, Rockport walking test & 1-mile | - | - | - | Yes | - |
| 14 | examen de aptitud | 0 | 0 | 0.00 € | Step test | Yes | - | - | Yes | Yes (step) |
| 15 | Multi Stage VO2 max Beep Test | 0 | 0 | 0.00 € | 20 meter shuttle run test | Yes | - | - | Yes | - |
| 16 | Ekblom-Bak Cycle Test | 0 | 0 | 0.00 € | Calculator | Yes | - | - | Yes | - |
| 17 | AF Fitness Assessment | 0 | 0 | 2.29 € | Calculator | Yes | - | - | - | - |
| 18 | BEEP TEST TEAM TRAINER | 0 | 0 | 4.49 € | 20 meter shuttle run test | Yes | | - | - | - |
| 19 | Bleep Test - Fitness Tests | 0 | 0 | 1.09 € | 20 meter shuttle run test, Pull Ups test, Press Ups test & Sit Ups test | - | - | - | Yes | - |
| 20 | 3 Minute Step Test - DIY Fitness Assessment | 0 | 0 | 5.49 € | 3 Minute Step Test | Yes | - | - | Yes | Yes (step) |
| 21 | Bleep,Test | 0 | 0 | 0.00 € | Standard Beep Test, YO YO Endurance Test | - | - | - | - | - |
| 22 | Beep Test Training Guide | 0 | 0 | 0.00 € | 20 meter shuttle run test | Yes | - | - | - | - |
| 23 | Treadmill Tracker | 0 | 0 | 3.49 € | - | - | Yes | Yes | Yes | - |
| 24 | Beep Fitness Test | 0 | 0 | 1.09 € | 15, 20 & 40 meter shuttle run test | Yes | - | - | Yes | - |
| 25 | UFT - Uber Fitness Test | 0 | 0 | 0.00 € | Cooper test, 300m run test | Yes | - | - | - | - |
| 26 | The Beep Test | 0 | 0 | 2.29 € | 20 meter shuttle run test, YO YO Endurance Test | Yes | - | - | Yes | - |
| 27 | FitnessMeter - Test & Measure | 0 | 0 | 2.29 € | 15 & 20 meter shuttle run test, Jump test, Abdominals test & Sprint test | Yes | - | - | Yes | - |
| 28 | 6MWT Lite | 0 | 0 | 0.00 € | 6 minutes walk test | - | - | - | - | - |
| 29 | 6MWT | 0 | 0 | 0.00 € | 6 minutes walk test | - | - | - | - | - |
| 30 | 6MWD | 0 | 1 | 0.00 € | 6 minutes walk test | - | - | - | - | - |
| 31 | The Beep Test Free | 0 | 0 | 0.00 € | 20 meter shuttle run test | Yes | - | - | Yes | - |

| | | | | | | | | | | |
|---|---|---|---|---|---|---|---|---|---|---|
| 32 | Fit Test | 0 | 0 | 3.49 € | Calculator | - | - | - | Yes | - |
| 33 | Beep Test | 0 | 0 | 3.49 € | 20 meter shuttle run test | Yes | - | - | Yes | - |
| 34 | Bleep Test Lite | 0 | 0 | 0.00 € | 20 meter shuttle run test | - | - | - | Yes | - |
| 35 | Bleep Test Solo | 0 | 0 | 1.09 € | 20m Bleep Test, Yo-Yo Intermittent Recovery, Yo-Yo Endurance and the official tests used by UK Police & Fire Services, | Yes | - | - | Yes | - |
| 36 | T4T - Test4Training | 0 | 0 | 0.00 € | - | - | - | - | Yes | - |
| 37 | Prueba Pacer VO2 Max Cardio y Shuttle Bleep | 0 | 0 | 5.49 € | 20 meter shuttle run test | Yes | - | - | Yes | - |
| 38 | VO2 Prueba De Pitidos Max - Pacer, Bleep & Shuttle | 0 | 0 | 0.00 € | 20 meter shuttle run test | - | - | - | Yes | - |
| 39 | BEEP TEST SLIM | 0 | 0 | 1.09 € | 20 meter shuttle run test | Yes | - | - | Yes | - |
| 40 | Music Beep Test - Pacer & Shuttle Run Fitness Test | 0 | 0 | 2.29 € | 15 & 20 meter shuttle run test | - | - | - | Yes | - |
| 41 | Shuttle Fit Trainer | 0 | 0 | 1.09 € | 20 meter shuttle run test | Yes | - | - | - | - |
| 42 | Test de Cooper | 0 | 0 | 0.00 € | Cooper Test | - | - | - | - | - |
| 43 | YO YO Endurance Test | 0 | 0 | 3.49 € | YO YO Endurance Test | Yes | - | - | - | - |
| 44 | Beep Test: Examen de Ejército Policía Militares | 0 | 0 | 0.00 € | 15 & 20 meter shuttle run test | Yes | - | - | - | - |
| 45 | Beep Test (Multi-Stage) | 0 | 0 | 1.09 € | 20 meter shuttle run test | Yes | - | - | - | - |
| 46 | VO2max Calculator | 0 | 0 | 0.00 € | Calculator | - | - | - | Yes | - |
| | **Total number of "Yes"** | | | | | 28 | 2 | 2 | 27 | 4 |

**"-" means the app doesn't contain the item**

**If the direct link does not work is because the app has been removed from the market**

**ELECTRONIC SUPPLEMENTARY MATERIAL** from the article by Muntaner-Mas et al. "A Systematic Review of Fitness Apps and Their Potential Clinical and Sports Utility for Objective and Remote Assessment of Cardiorespiratory Fitness"

**Table 8,** Qualitative assessment of fitness apps stored in App Store (iTunes) (Continued),

| | App name | Historic measurements | Export results | Prompt self-monitoring | Social Network | Reference values | Scientific validation | Multiple users | Language | Update date | Sum of "Yes" |
|---|---|---|---|---|---|---|---|---|---|---|---|
| 1 | HRV4Training | Yes | Yes | Yes | - | - | Yes | - | English & Italian | 26/09/2018 | 7 |
| 2 | MyHeart Counts | Yes | Yes | Yes | - | Yes | - | - | English | 09/07/2018 | 7 |
| 3 | Fitness Test pro | Yes | Yes | - | Yes | Yes | - | Yes | English | 19/09/2017 | 7 |
| 4 | AeroExaminer - Aerobic VO2 Max Test & Conditioning | Yes | Yes | Yes | Yes | - | - | Yes | English | 10/01/2017 | 8 |
| 5 | CardioCoach | - | - | - | - | - | - | - | English | 04/10/2017 | 0 |
| 6 | iWalkAssess | Yes | Yes | - | Yes | Yes | Yes | - | English | 05/20/2018 | 6 |
| 7 | Team Bleep Test | Yes | Yes | - | Yes | Yes | - | Yes | English | 26/07/2017 | 5 |
| 8 | CycleCoach - Fitness Test | Yes | - | Yes | Yes | - | - | - | English & Japanese | 06/05/2016 | 4 |
| 9 | Multi Stage Pacer Test | - | - | - | Yes | - | - | - | English | 27/09/2017 | 2 |
| 10 | Police Fitness | Yes | - | - | - | - | - | - | English | 13/10/2017 | 2 |
| 11 | Bleep Test Pro - 15m & 20m - Beep Test Shuttle Run | Yes | - | - | Yes | - | - | Yes | English | 13/10/2017 | 3 |
| 12 | BEEP TEST TRAINER | Yes | Yes | - | Yes | - | - | Yes | English | 25/01/2017 | 6 |
| 13 | Max O2 | Yes | - | - | - | Yes | - | - | English | 14/12/2011 | 3 |
| 14 | examen de aptitud | - | - | - | Yes | Yes | - | - | English | 01/04/2014 | 4 |
| 15 | Multi Stage VO2 max Beep Test | Yes | Yes | - | Yes | Yes | - | Yes | Spanish & English | 20/08/2018 | 7 |
| 16 | Ekblom-Bak Cycle Test | - | Yes | - | - | Yes | - | - | English & Swedish | 07/11/2017 | 4 |
| 17 | AF Fitness Assessment | - | - | - | - | Yes | - | - | English | 15/01/2018 | 3 |
| 18 | BEEP TEST TEAM TRAINER | Yes | Yes | - | - | - | - | Yes | English | 25/01/2017 | 4 |
| 19 | Bleep Test - Fitness Tests | Yes | - | - | - | - | - | - | English, French & Portuguese | 27/04/2017 | 2 |
| 20 | 3 Minute Step Test - DIY Fitness Assessment | - | Yes | - | Yes | Yes | - | - | English | 09/02/2017 | 5 |
| 21 | Bleep,Test | Yes | - | - | - | - | - | Yes | English | 27/10/2018 | 2 |
| 22 | Beep Test Training Guide | - | - | - | - | Yes | - | - | English | 24/11/2015 | 2 |
| 23 | Treadmill Tracker | Yes | Yes | - | - | - | - | - | English | 20/11/2015 | 3 |
| 24 | Beep Fitness Test | Yes | - | - | - | Yes | - | - | English | 08/09/2017 | 4 |
| 25 | UFT - Uber Fitness Test | - | - | - | - | Yes | - | - | English | 03/08/2015 | 2 |
| 26 | The Beep Test | - | - | - | Yes | - | - | Yes | English | 05/10/2014 | 4 |
| 27 | FitnessMeter - Test & Measure | - | Yes | - | - | Yes | - | Yes | English | 21/02/2018 | 6 |
| 28 | 6MWT Lite | Yes | Yes | - | Yes | - | - | - | Spanish, German & English | 11/10/2013 | 3 |
| 29 | 6MWT | Yes | Yes | - | Yes | - | - | - | Spanish, German & English | 07/11/2013 | 3 |
| 30 | 6MWD | Yes | Yes | - | Yes | - | - | - | Spanish, German & English | 14/05/2017 | 3 |
| 31 | The Beep Test Free | - | - | - | Yes | - | - | Yes | English | 16/09/2014 | 4 |

| | | | | | | | | | | | |
|---|---|---|---|---|---|---|---|---|---|---|---|
| 32 | Fit Test | - | Yes | - | Yes | - | - | - | English | 17/10/2014 | 3 |
| 33 | Beep Test | Yes | Yes | - | - | - | - | Yes | English | 22/02/2017 | 5 |
| 34 | Bleep Test Lite | - | - | - | - | - | - | - | English | 28/07/2017 | 1 |
| 35 | Bleep Test Solo | Yes | Yes | - | - | - | - | Yes | English | 26/07/2017 | 5 |
| 36 | T4T - Test4Training | Yes | Yes | - | - | Yes | - | - | Spanish & English | 18/01/2018 | 4 |
| 37 | Prueba Pacer VO2 Max Cardio y Shuttle Bleep | Yes | Yes | - | Yes | - | - | Yes | Spanish & English | 16/03/2017 | 6 |
| 38 | VO2 Prueba De Pitidos Max - Pacer, Bleep & Shuttle | Yes | - | - | - | - | - | - | English | 16/03/2017 | 2 |
| 39 | BEEP TEST SLIM | Yes | Yes | - | - | - | - | - | English | 25/01/2017 | 4 |
| 40 | Music Beep Test - Pacer & Shuttle Run Fitness Test | - | - | - | - | Yes | - | - | English | 08/05/2014 | 2 |
| 41 | Shuttle Fit Trainer | Yes | - | - | - | - | - | - | English | 30/11/2016 | 2 |
| 42 | Test de Cooper | - | - | - | - | Yes | - | - | English | 15/09/2016 | 1 |
| 43 | YO YO Endurance Test | - | - | - | - | - | - | - | English | 24/10/2018 | 1 |
| 44 | Beep Test: Examen de Ejército Policía Militares | - | - | - | - | - | - | - | Spanish, German, French, English, | 22/06/2017 | 1 |
| 45 | Beep Test (Multi-Stage) | - | - | - | - | - | - | - | English | 20/02/2018 | 1 |
| 46 | VO2max Calculator | - | - | - | - | - | - | - | English | 08/04/2015 | 1 |
| | **Total number of "Yes"** | 27 | 22 | 4 | 18 | 17 | 2 | 14 | | | |

**"-" means the app doesn't contain the item**

**If the direct link does not work is because the app has been removed from the market**

**ELECTRONIC SUPPLEMENTARY MATERIAL** from the article by Muntaner-Mas et al. “A Systematic Review of Fitness Apps and Their Potential Clinical and Sports Utility for Objective and Remote Assessment of Cardiorespiratory Fitness”

**Table 9,** Qualitative assessment of fitness apps stored in Google Play,

| | App name | Score | Ratings | Downloads | Price | Test mode | Test instructions | HR | GPS | $VO_2$ estimation | External devices needed |
|---|---|---|---|---|---|---|---|---|---|---|---|
| 1 | HRV4Training | 4.4 | 329 | 5,000+ | 9.99 € | Published algorithms | Yes | Yes | - | Yes | - |
| 2 | Fitness Test pro | 4 | 67 | 1,000+ | 2.41 € | Coopers 12-min run, Bruce treadmill test, Any distance run test, Beep-test (Yo-Yo endurance test, The Andersen test, Watt-max | Yes | - | - | Yes | - |
| 3 | iWalkAssess | - | - | 100+ | 0.00 € | 10m walk test and 6-min walking test | Yes | - | - | - | - |
| 4 | Bruce Treadmill Test Lite | 3.4 | 8 | 1,000+ | 0.00 € | Bruce treadmill test | - | - | - | Yes | - |
| 5 | Bruce Treadmill Test Protocol | 0 | 0 | 10+ | 4.99 € | Bruce treadmill test | Yes | - | - | Yes | - |
| 6 | Physical V02 Fitness Beep Test | 3.8 | 16 | 500+ | 4.99 € | 20 meter shuttle run test | Yes | - | - | Yes | - |
| 7 | Marine Corps PT Calculator - USMC Physical | 3.8 | 19 | 5,000+ | 0.00 € | 3 mile run, MC Combat fitness test, MC physical fitness test | - | - | - | - | - |
| 8 | TOHRC Walk Test | 3.5 | 2 | 100+ | 0.00 € | 2 and 6-min walking test | - | - | - | - | Yes |
| 9 | 3 Minute Step Test | 4 | 5 | 500+ | 6.49 € | YMCA step test | Yes | - | - | Yes | - |
| 10 | blp - The Bleep Test App | 3.5 | 67 | 10,000+ | 0.00 € | 20 meter shuttle run test | Yes | - | - | Yes | - |
| 11 | Functional Assessment + G-Code | 4.8 | 22 | 500+ | 0.79 € | 6-min walking test | Yes | - | - | - | - |
| 12 | Run U2 | 4.4 | 76 | 5,000+ | 0.00 € | 7 minutes, Astrand-treadmill, Balke-track, Balke treadmill, Bruce- | Yes | - | - | Yes | - |
| 13 | Fitness Assessments | 3.7 | 12 | 500+ | 3.34 € | Rockport test | - | - | - | Yes | - |
| 14 | Test de Léger | 4.2 | 331 | 50,000+ | 0.00 € | 20 meter shuttle run test | Yes | - | - | - | - |
| 15 | 12 Minute Vo2 Max Run Test | 3.8 | 65 | 10,000+ | 0.00 € | Cooper Test | Yes | - | Yes | Yes | - |
| 16 | Physical Fitness V02 Beep Test | 3.9 | 283 | 50,000+ | 0.00 € | 20 meter shuttle run test | Yes | - | - | Yes | - |
| 17 | Shuttle Run VO2Max Pacer Test | 3.8 | 78 | 10,000+ | 0.00 € | 15 meter shuttle run test | Yes | - | - | Yes | - |
| 18 | Calculadora Funcional Trainer | 4.6 | 33 | 1,000+ | 0.00 € | 1,5 miles test | - | - | - | Yes | - |
| 19 | Beep Test Pro | 4 | 45 | 5,000+ | 0.99 € | 20 meter shuttle run test | - | - | - | Yes | - |
| 20 | Yo-Yo Intermittent Pro | 3.9 | 21 | 1,000+ | 0.99 € | 20 meter shuttle run test | - | - | - | Yes | - |
| 21 | Test de marcha de 6 minutos | 4.1 | 64 | 5,000+ | 0.00 € | 6-min walking test | - | - | - | Yes | - |
| 22 | Beep Test Calculator | 4.5 | 2 | 1,000-5,000 | 0.00 € | 20 meter shuttle run test | - | - | - | Yes | - |
| 23 | Bruce Protocol Stress Calci | 1 | 1 | 100+ | 0.00 € | Bruce treadmill test | - | - | - | Yes | - |
| 24 | Army Fitness Calculator - APFT | 2.7 | 3 | 100 - 500 | 0.00 € | 2 mile run | - | - | - | - | - |
| 25 | Cardio Exercise Prescription | 4.4 | 5 | 1,000+ | 0.00 € | 6MWT | - | - | - | Yes | - |
| 26 | Astrand Cycle Fitness Calci | - | - | 100+ | 0.00 € | Astrand Cycle Fitness Test | - | - | - | Yes | - |
| 27 | VO2 Max Calculator | 1 | 1 | 500+ | 0.00 € | | - | - | - | Yes | - |
| 28 | FitCal - Fitness Calculators | 4.5 | 12 | 500+ | 0.75 € | - | - | - | - | Yes | - |
| 29 | Test VMA Pro | 3 | 55 | 10,000+ | 0.00 € | Test piste Léger-Boucher | Yes | - | - | - | - |
| 30 | V02 Calculadora | 2.8 | 4 | 1,000+ | 0.00 € | Rockport Walking test | Yes | - | - | Yes | - |
| 31 | eRunners | 5 | 1 | 5+ | 1.49 € | Test de Cooper | Yes | - | Yes | Yes | - |
| 32 | Prueba Beep | 3.8 | 723 | 100,000+ | 0.00 € | 20 meter shuttle run test | - | - | - | Yes | - |

| | | | | | | | | | | | |
|---|---|---|---|---|---|---|---|---|---|---|---|
| 33 | Yo-Yo Test Intermitente | 4.5 | 243 | 50,000+ | 0.00 € | 20 meter shuttle run test | - | - | - | Yes | - |
| 34 | AG-Evaluapp - Fitness Tests | 4.7 | 15 | 1,000+ | 0.00 € | Course Navette, Yo-yo test | - | - | - | Yes | - |
| 35 | FitCalc+ Fitness & Health Calculator - Gym Tools | 5 | 5 | 500+ | 0.00 € | | - | - | - | Yes | - |
| 36 | 2,4 Km Run Test (Fitness Test) | - | - | 100+ | 0.00 € | Test de Cooper | - | - | - | Yes | - |
| 37 | Beep Test vocacional Ejército | 3.9 | 533 | 100,000+ | 0.00 € | 20 meter shuttle run test | - | - | - | - | - |
| 38 | Fitga Fitness Tracker | 4 | 10 | 500+ | 0.00 € | Rockport Fitness Walk Test and Treadmill test | Yes | - | - | Yes | - |
| 39 | Fitness Test | 3.8 | 40 | 10,000+ | 0.00 € | 20 meter shuttle run test | - | - | - | Yes | - |
| 40 | 12 Minute Run Test (Cooper Test) | 5 | 4 | 500+ | 0.00 € | Test de Cooper | Yes | - | - | Yes | - |
| 41 | Astrand Treadmill Test (Vo2max Test) | 5 | 1 | 100+ | 0.00 € | Astrand Treadmill Test | Yes | - | - | Yes | - |
| 42 | Balke Vo2max Test | 5 | 1 | 100+ | 0.00 € | Balke VO2max test | Yes | - | - | Yes | - |
| | **Total number of "Yes"** | | | | | | 21 | 1 | 2 | 34 | 1 |

**"-" means the app doesn't contain the item**

**If the direct link does not work is because the app has been removed from the market**

**ELECTRONIC SUPPLEMENTARY MATERIAL** from the article by Muntaner-Mas et al. "A Systematic Review of Fitness Apps and Their Potential Clinical and Sports Utility for Objective and Remote Assessment of Cardiorespiratory Fitness"

**Table 9,** Qualitative assessment of fitness apps stored in Google Play (Continued),

| | App name | Historic measurements | Export results | Prompt self-monitoring | Social Network | Reference values | Scientific validation | Multiple users | Update date | Sum of "Yes" |
|---|---|---|---|---|---|---|---|---|---|---|
| 1 | HRV4Training | Yes | ,csv Format | Yes | - | - | Yes | - | 10/10/2018 | 7 |
| 2 | Fitness Test pro | Yes | Yes | - | Yes | Yes | - | Yes | 18/03/2013 | 7 |
| 3 | iWalkAssess | Yes | - | Yes | - | Yes | - | Yes | 22/08/2018 | 5 |
| 4 | Bruce Treadmill Test Lite | - | - | - | Yes | - | - | Yes | 04/05/2014 | 3 |
| 5 | Bruce Treadmill Test Protocol | Yes | - | - | Yes | - | - | Yes | 04/05/2014 | 5 |
| 6 | Physical V02 Fitness Beep Test | Yes | - | - | Yes | Yes | - | Yes | 11/01/2017 | 6 |
| 7 | Marine Corps PT Calculator - USMC | Yes | - | - | - | - | - | - | 23/05/2017 | 1 |
| 8 | TOHRC Walk Test | Yes | ,csv Format, ,pdf | - | - | - | Yes | - | 03/08/2017 | 3 |
| 9 | 3 Minute Step Test | - | - | - | Yes | Yes | - | - | 01/04/2014 | 4 |
| 10 | blp - The Bleep Test App | Yes | - | - | - | - | - | Yes | 04/02/2014 | 4 |
| 11 | Functional Assessment + G-Code | - | - | - | - | Yes | - | - | 24/10/2018 | 2 |
| 12 | Run U2 | - | - | - | - | - | - | - | 18/02/2017 | 2 |
| 13 | Fitness Assessments | Yes | ,xml Format | - | Yes | - | - | Yes | 09/03/2015 | 5 |
| 14 | Test de Léger | - | - | - | - | - | - | - | 15/06/2015 | 1 |
| 15 | 12 Minute Vo2 Max Run Test | Yes | - | - | Yes | Yes | - | - | 12/01/2017 | 6 |
| 16 | Physical Fitness V02 Beep Test | Yes | - | - | Yes | Yes | - | - | 11/01/2017 | 5 |
| 17 | Shuttle Run VO2Max Pacer Test | Yes | - | - | Yes | Yes | - | - | 11/01/2017 | 5 |
| 18 | Calculadora Funcional Trainer | - | - | - | - | - | - | - | 21/08/2017 | 1 |
| 19 | Beep Test Pro | Yes | Yes | - | Yes | - | - | Yes | 25/10/2018 | 5 |
| 20 | Yo-Yo Intermittent Pro | Yes | ,csv Format | - | Yes | - | - | Yes | 26/10/2018 | 5 |
| 21 | Test de marcha de 6 minutos | Yes | - | - | Yes | - | - | Yes | 09/10/2013 | 4 |
| 22 | Beep Test Calculator | - | - | - | - | - | - | - | 03/10/2018 | 1 |
| 23 | Bruce Protocol Stress Calci | - | - | - | - | - | - | - | 01/10/2018 | 1 |
| 24 | Army Fitness Calculator - APFT | Yes | - | - | - | - | - | - | 27/12/2016 | 1 |
| 25 | Cardio Exercise Prescription | - | - | - | - | - | - | - | 16/06/2014 | 1 |
| 26 | Astrand Cycle Fitness Calci | - | - | - | - | Yes | - | - | 23/10/2018 | 2 |
| 27 | VO2 Max Calculator | - | - | - | - | Yes | - | - | 27/06/2017 | 2 |
| 28 | FitCal - Fitness Calculators | - | - | - | - | - | - | - | 17/10/2013 | 1 |
| 29 | Test VMA Pro | Yes | - | - | - | - | - | Yes | 20/09/2017 | 3 |
| 30 | V02 Calculadora | - | - | - | - | Yes | - | - | 25/08/2017 | 3 |
| 31 | eRunners | - | - | - | - | - | - | - | 17/11/2013 | 3 |
| 32 | Prueba Beep | - | - | - | - | - | - | - | 25/10/2018 | 1 |
| 33 | Yo-Yo Test Intermitente | - | - | - | - | - | - | - | 26/10/2018 | 1 |

| | | | | | | | | | | |
|---|---|---|---|---|---|---|---|---|---|---|
| 34 | AG-Evaluapp - Fitness Tests | Yes | - | - | Yes | - | - | Yes | 24/08/2017 | 4 |
| 35 | FitCalc+ Fitness & Health Calculator - Gym | - | - | - | - | - | - | - | 29/06/2018 | 1 |
| 36 | 2,4 Km Run Test (Fitness Test) | Yes | - | - | - | - | - | Yes | 04/06/2018 | 3 |
| 37 | Beep Test vocacional Ejército | - | - | - | - | - | - | - | 29/01/2016 | 0 |
| 38 | Fitga Fitness Tracker | Yes | - | - | - | - | - | - | 30/04/2015 | 3 |
| 39 | Fitness Test | - | - | - | - | - | - | - | 03/01/2014 | 1 |
| 40 | 12 Minute Run Test (Cooper Test) | - | - | - | - | Yes | - | Yes | 14/06/2018 | 4 |
| 41 | Astrand Treadmill Test (Vo2max Test) | - | - | - | - | Yes | - | Yes | 15/06/2018 | 4 |
| 42 | Balke Vo2max Test | - | - | - | - | Yes | - | Yes | 15/06/2018 | 4 |
| | **Total number of "Yes"** | 20 | 6 | 2 | 13 | 14 | 2 | 16 | | |

**"-" means the app doesn't contain the item**
**If the direct link does not work is because the app has been removed from the market**
